# Vasopressin-related copeptin is a novel predictor of early endothelial dysfunction in patients with adult polycystic kidney disease

**DOI:** 10.1186/s12882-016-0406-4

**Published:** 2016-11-30

**Authors:** Ismail Kocyigit, Mahmut Ilker Yilmaz, Ozkan Gungor, Eray Eroglu, Aydin Unal, Ozcan Orscelik, Bulent Tokgoz, Murat Sipahioglu, Ahmet Sen, Juan Jesús Carrero, Oktay Oymak, Jonas Axelsson

**Affiliations:** 1 Department of Nephrology, Erciyes University Medical Faculty, Kayser, Turkey; 2 Department of Nephrology, Gülhane School of Medicine, Ankara, Turkey; 3 Department of Nephrology, KahramanMaras Sutcu Imam University, Kahramanmaras, Turkey; 4 Department of Cardiology, Mersin University Medical Faculty, Mersin, Turkey; 5 Department of Biochemistry, Erciyes University Medical Faculty, Kayseri, Turkey; 6 Division of Renal Medicine, Department of Clinical Science, Karolinska Institutet, Stockholm, Sweden; 7 Vascular Biology Group, Department of Medical Biochemistry and Biophysics, Karolinska Institutet, Stockholm, Sweden; 8 Department of Clinical Immunology, C2:66, Karolinska University Hospital, 14186 Stockholm, Sweden

**Keywords:** Endothelial dysfunction, Arterial dysfunction, Hereditary, CVD risk, AVP

## Abstract

**Background:**

In this study, we examined the relative usefulness of serum copeptin levels as a surrogate marker of vasopressin (AVP) in adult polycystic kidney disease (ADPKD) by correlating it with baseline and longitudinal changes in markers of both renal function and common CVD manifestations (hypertensive vascular disease, atherosclerosis and endothelial dysfunction) that accompany the progression of this disease.

**Methods:**

We studied a cohort of young and otherwise healthy ADPKD patients (*n* = 235) and measured cardiovascular function using flow-mediation dilatation (FMD), carotid intima media thickness (cIMT) and pulse wave velocity (PWV), as well as serum copeptin (commercial ELISA, a stable marker of AVP activity). The same analyses were carried out at baseline and after 3 years of follow-up.

**Results:**

At baseline, median eGFR was 69 mL/min./1.73 m^2^, mean FMD 6.9 ± 0.9%, cIMT 0.7 ± 0.1 mm, and PWV 8.1 ± 1.2 m/s. At follow-up, equivalent values were 65 (44–75) mL/min./1.73 m^2^, 5.8 ± 0.9%, 0.8 ± 0.1 mm. and 8.2 ± 1.3 m/s. with all changes statistically significant. Plasma copeptin also rose from 0.62 ± 0.12 to 0.94 ± 0.19 ng/mL and this change correlated with ΔeGFR (-0.33, *p* < 0.001), ΔFMD (0.599, *p* < 0.001), ΔcIMT (0.562, *p* < 0.001) and ΔPWV (0.27, *p* < 0.001) also after linear regression modeling to correct for confounders. Finally, ROC analysis was done for a high baseline copeptin with ΔeGFR [cut-off:≤59], ΔFMD [cut-off: ≤7.08], ΔcIMT [cut-off:>0.76], and ΔPWV [cut-off:≤7.80].

**Conclusions:**

Vascular dysfunction as reflected by FMD and cIMT, but not PWV or an altered cardiac geometry, precede most other signs of disease in ADPKD but is predicted by elevated levels of the circulating AVP-marker copeptin.

## Background

Autosomal dominant polycystic kidney disease (ADPKD) is the most frequently hereditary cause of renal failure but also an important cause of hypertension and cardiovascular diseases (CVD). Renal and extra-renal cystic manifestations are the main characteristics of a disease that often leads to the need for renal replacement therapy by the sixth decade of life. The risk of CVD morbidity and mortality are both highly elevated in ADPKD as compared to the general population, but it is not known if this is mainly a consequence of the disease itself or if it is linked primarily to the drop in renal function [1, 2].

Recent advances have linked cyst formation in ADPKD to arginine-vasopressin hormone (AVP)-signalling through the vasopressin V2-receptor and subsequent phosphodiesterase-driven cAMP modulation of that signal [3, 4]. Clinically, the V2-antagonist tolvaptan was recently shown to slow the increase in total kidney volume and the decline in kidney function over a 3-year trial period [5].

Measuring AVP directly is difficult, as over 90% is tightly bound to platelets [6]. Copeptin is a 39 amino acid glycopeptides which forms the C-terminal part of the AVP-precursor pre-provasopressin [7]. Activation of the AVP-system drives copeptin secretion from the posterior pituitary gland into the circulation in equimolar amounts with AVP [8, 9]. Plasma copeptin is elevated in patients with autosomal dominant polycystic kidney disease and predicts disease progression [10], but does not appear to be heavily influenced by GFR [11].

In this study, we examined the relative usefulness of serum copeptin levels as a surrogate marker of AVP by correlating it with baseline and longitudinal changes in markers of both renal function and common CVD manifestations (hypertensive vascular disease, atherosclerosis and endothelial dysfunction) that accompany the progression of ADPKD.

## Methods

### Patients, ethics, consent and permissions

Between March 2012 and March 2015, all 235 ADPKD patients with normal renal function and followed at either Kayseri Erciyes University School of Medicine or the Ankara Gulhane School of Medicine (identified through the Turkish Society of Nephrology’s Polycystic Kidney Disease Working Group Registry) were screened for inclusion in the study. Prior approval for the study had been obtained from the local ethics committees at both hospitals. Eligible patients were invited to enroll in the study and, following verbal and written information about the study, asked to give written consent. Only patients that gave written, informed consent to participate in the study were recruited, and the study conformed to the Declaration of Helsinki as amended. The study was prepared according to the STROBE guidelines/methodology.

In enrolled patients, the diagnosis of ADPKD was re-established based on clinical data, family history and a new ultrasound of the kidneys using the criteria described by Pei et al [12]. Demographic characteristics (e.g. sex, age, education and smoking history), renal disease symptoms (e.g. history of hematuria, urinary tract infections, kidney stone, etc.) and cardiovascular manifestations (e.g. hypertension and mitral valve prolapse) were recorded using a web-based data collection form. We excluded patients prescribed drugs likely to affect copeptin (eg. loop diuretics (*n* = 7), SSRIs (*n* = 4), NSAIDs (*n* = 6), demeclocycline (*n* = 1), statins (*n* = 10), clofibrate (*n* = 2), chlorpromazine (*n* = 1), and vasopressin analogues(*n* = 3). In the end, the study cohort comprised a total of 202 ADPKD patients with normal renal function. At 36 months after the initial evaluation, patients were recalled and asked to undergo the same procedures a second time. Four patients did not complete follow-up, but their data were kept for baseline analyses.

### Ambulatory blood pressure

Blood pressure monitoring over 24 h was performed using a Del Mar Medical Ressurometer Model P6 (Del Mar Reynolds, Irvine, CA, USA) together with the manufacturer’s software. Ambulatory measurements were conducted once every 15min from 7 am until 11 pm, and once every 30min from 11 pm until 7 am. Data were summarized using the mean values for day and night. Hypertension was considered to be present if the average systolic pressure was ≥130 mmHg and/or the average diastolic pressure was ≥80 mmHg for whole day, or if the individual was taking any of the allowed antihypertensive medications.

### Biochemical analyses

Venous blood was drawn following a 12 h self-reported fast in the morning due to circadian rhytmicity of AVP. Glucose, creatinine, and lipids were assessed using standard methods. Estimated GFR (eGFR) was calculated using the Chronic Kidney Disease Epidemiology Collaboration (CKD-EPI) equation [13]. High sensitivity C-reactive protein (hs-CRP) was measured using a BN2 model nephelometer (Dade-Behring, Germany). Copeptin was measured in serum using an ELISA kit from Phoenix Pharmaceuticals (California, USA, cat. no. EK-065-32). The reported intra- and inter-assay coefficients of variation (CVs) reported by the manufacturer were <10 and <15% respectively.

### Echocardiography

All participants were examined using a Vivid 7 instrument (GE Medical Systems, Milwaukee, WI, USA) with a 2.5-MHz transducer and harmonic imaging. The echocardiographies were all performed by a specialist in echocardiography and according to the recommendations of the American Society of Echocardiography. Briefly, at least three consecutive beats in sinus rhythm were recorded, and the average values used. The LV end-diastolic and end-systolic dimensions (LVEDD and LVESD) and interventricular septal and posterior wall thicknesses (IVSd and LPWd) were measured from M-mode images of the left ventricle generated in the long-axis view with the cursor at the tip of the mitral valve leaflets. The LV ejection fraction was calculated using the formula: LVEF % = (LVEDV − LVESV)/LVEDV × 100. The left ventricular mass (LVM) was calculated using the formula: LVM = 0.8 × (1.04 [(IVSd + LVEDD + LPWd) ^3^ − (LVEDD) ^3^)) + 0.6 g [14].

### Endothelial function test

Endothelial dysfunction was assessed according to the transient ischemia method described by Celemajer et al. [15]. Measurements were made by a single observer using an ATL 5000 ultrasound system (Advanced Technology Laboratories Inc., Bothell, WA, USA) using a 12-Mhz probe. Three adjacent measurements of end-diastolic brachial artery diameter were made from single 2D frames. The maximum flow-mediated vasodilation (FMD) diameter was calculated by averaging three consecutive measurements, and FMD was then calculated as the percentage change in diameter compared with baseline. All images were recorded for subsequent blinded analysis.

### Thickness of the carotid artery intima-media (c-IMT)

Ultrasonographic studies on common carotid artery was done on both sides and using high-resolution Doppler ultrasound (ATL 5000) with a 5–12 MHz linear transducer. A single blinded operator performed all measurents on two stored longitudinal images of each artery. The four values were averaged to calculate mean c-IMT.

### Pulse wave velocity

Pulse wave analysis was performed twice on each side, in the carotid and femoral arteries. We used a machine (Micro Medical Pulse Trace, Rochester, UK) in accordance with the manufacturer’s recommendations. PWV was calculated automatically by measuring the time for the pulse wave to travel between the carotid and femoral arteries. All measurements were performed over 15 heart beats by a single operator.

### Statistical analysis

Shapiro-Wilk’s test, histogram and q-q plots were examined to assess normal distribution. Levene test was performed to assess the variance in homogenity. The statistical significance of differences between groups were assessed by two-way paired t- test (normally distributed data) or Wilcoxon t- test (non-normal distribution). In multivariate analyses, all variables for which a unadjusted, univariate linear regression analysis showed a *p*-value <0.10 were included in a backward elimination multivariate linear regression analysis, with the remaining variables compared as concerns risks by using the likelihood ratio tests. *p* < 0.05 was considered significant, and the confidence interval (CI) was set to 95%. All statistical analyses were performed using SPSS version 15 (SPSS,Inc., Chicago, Ill., USA).

## Results

### Patient characteristics at baseline and follow-up

**Fig. 1:**
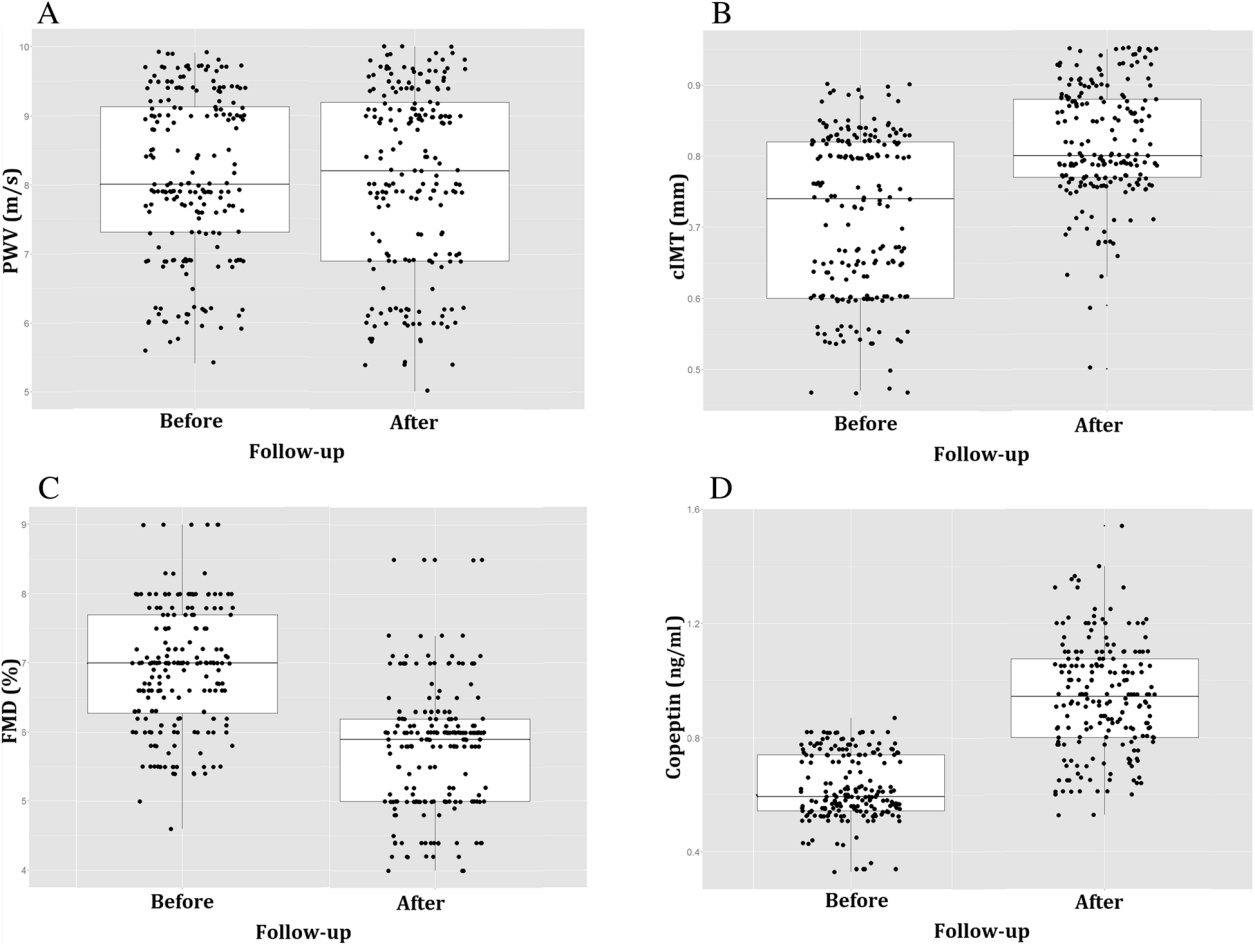
Comparison of arterial function markers and circulating copeptin values at baseline and after 36 months in 202 ADPKD patients. PWV, pulse-wave velocity. CIMT, carotid artery intima media thickness. FMD, post-ischemia flow mediated vasodilation

Demographical features of the 202 ADPKD patients are summarized in Table 1. Baseline biochemical, echocardiographic and other recorded data along with those obtained after 3 years are given in Table 2. As expected, there was a small but statistically significant decrease in eGFR (69[54–78] to 65[44–75] mL/min/1.73 m^2^; *p* < 0.001) and hemoglobin during follow-up, while serum phosphorus, HDL- and LDL- cholesterol, plasma glucose, average 24-h systolic and diastolic blood pressure (SBP and DBP), and proteinuria all rose. Of the studied markers of cardiovascular function, LVM (1.1%; *p* < 0.001), PWV (2%; *p* < 0.001), cIMT (14%; *p* < 0.001), copeptin (52%; *p* < 0.001) and hs-CRP (165%; *p* < 0.001) all increased during follow-up, while FMD (-16%; *p* < 0.001) decreased (Table 2, Fig. 1). There were no statistically significant changes in LVM, LVEF or LVED.

**Table 1:** Baseline clinical characteristic of the 202 ADPKD patients

Variable	n(%)
Age (years)	34.86 ± 9.17
Gender (male)	105(52)
Hypertension (present)	70(35.0)
Smokers	21(10.3)
Diabetes(present)	11(5)
Receiving oral hypoglycemictherapy	10(5)
With cardiovascular comorbidities	7(3)

Values are expressed as mean ± standard deviation or frequency (percentage)

**Table 2:** Comparison of clinical, echocardiographic features and biochemical data before and after 3 year follow-up

Variable	Reference values	Baseline (*n* = 202)	Follow-up (*n* = 202)	ΔChange%	*p*
eGFR(ml/min/1.73 m^2^)	>90	69.0(54.0–78.0)	65.0(44.0–75.0)	−5.80	**<0.001**
BMI (kg/m^2^)	20–24	24.67 ± 2.78	24.98 ± 2.47	1.26	0.212
Calcium (mg/dl)	8.8–10.2	8.27 ± 0.45	8.19 ± 0.31	−0.97	**0.039**
Sodium (mmol/L)	(135–144)	139.48 ± 4.22	137.59 ± 3.62	−1.37	0.066
Phosphorus (mg/dl)	2.5–4.5	4.80(4.13–5.30)	5.70(4.80–8.20)	18.75	**<0.001**
HDL Cholesterol (mg/dl)	45–65	43(39.0–46.0)	45.0(40.25–47.00)	4.65	**0.003**
LDL Cholesterol (mg/dl)	100–130	123(118–129)	128(109–139)	4.07	**0.005**
Total Cholesterol (mg/dl)	70–200	193.71 ± 15.02	194.05 ± 16.44	0.18	0.843
Triglyceride (mg/dl)	40–130	140.37 ± 11.41	134.46 ± 11.19	−4.21	**<0.001**
Hemoglobin (g/l)	12–16	14.0(12.8–14.7)	13.6(13.1–14.0)	−2.86	**0.002**
Glucose (mg/dl)	82–115	85.0(78–120)	88(80–115)	3.53	**0.001**
Urine volume (mL)	-	1868 ± 572	1780 ± 568	−3.71	<0.001
U-osmolarity (mOsm/kg)	-	205(178–245)	289(254–395)	42.85	<0.001
Average 24-h systolic BP, mmHg	<130	135 ± 6.9	142 ± 7.2	5.19	**<0.001**
Average 24-h diastolic BP, mmHg	<80	83 ± 4.8	86 ± 5.3	3.61	**<0.001**
Proteinuria (mg/day)	0–150	1100 (500–1870)	1540 (720–2600)	40.00	**<0.001**
Left ventricular ejection fraction(%)	>55	64.4 ± 5.0	62.1 ± 5.8	−3.57	0.245
Left ventricular mass (g)	49–115	169.4 ± 37.3	171.3 ± 46.5	1.12	0.311
Left ventricular end-diastolic diameter (mm)	42–59	46.5 ± 2.9	47.0 ± 3.4	0.88	0.175
PWV (m/sec)	-	8.10 ± 1.20	8.24 ± 1.34	1.73	**0.044**
FMD, %	-	6.92 ± 0.87	5.78 ± 0.92	−16.47	**<0.001**
CIMT(mm)	-	0.72 ± 0.11	0.82 ± 0.08	13.89	**<0.001**
Copeptin (ng/mL)	N/A	0.62 ± 0.12	0.94 ± 0.19	51.61	**<0.001**
Hs-CRP (mg/l)	<0.2	3.40(2.20–4.0)	9.00(5.10–19.0)	164.71	**<0.001**

Values are expressed as mean ± standard deviation or median(1^st^–3^rd^quartiles)

*p* value below 0.05 was considered significant and significant parameters were shown by bold typ﻿e

ΔChange (%) : (follow up value- baseline value)/baseline value*100

*eGFR*, estimated glomerular filtration rate, *BMI* Body massindex, *HDL-C* High density lipoprotein cholesterol, *LDL-C* Low density lipoprotein cholesterol, *Hs-CRP* High sensitivity C-reactive protein, *PWV* Pulse wave velocity, *c-IMT* carotid artery intima-media thickness

### Univariate correlations and regression

**Fig. 2:**
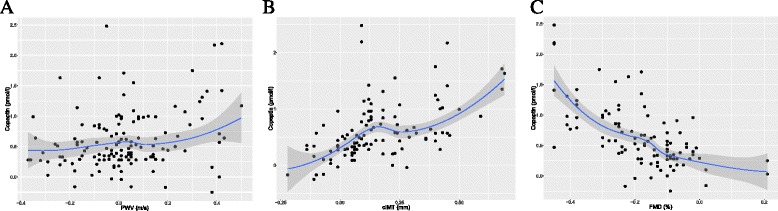
Univariate correlations between serum copeptin with PWV, CIMT and FMD in 202 young ADPKD patients with well-preserved renal function

At baseline, Spearman Rank analysis showed significant correlations between serum copeptin and proteinuria (rho = 0.35; *p* < 0.01), mean 24 h systolic (rho = 0.17; *p* < 0.05) and diastolic (rho = 0.22; *p* < 0.01) blood pressures, cIMT (rho = 0.63; *p* < 0.001), eGFR (rho = -0.30, *p* < 0.001) and urine osmolality (rho = 0.43; *p* < 0.001). We next performed univariate regression analysis to assess correlations between changes in copeptin over time with Δ PWV (*r* ^2^ = 0.03; *p* < 0.05), Δ cIMT (*r* ^2^ = 0.32; *p* < 0.01) and Δ FMD (*r* ^2^ = 0.39; *p* < 0.001) (Tables 3 and 4 and Fig. 2).

**Table 3:** Spearman Rank correlation matrix showing univariate correlates of baseline copeptin with selected variables at baseline

Variable	S-Copeptin (ng/mL)	Proteinuria (mg/day)	SBP (mmHg)	DBP (mmHg)	U-osmolarity (mOsm/kg)	U-volume (mL)	cIMT (mm)	LVM (g)	LVEDD (mm)	eGFR (ml/min/1.73 m^2^)
S-Copeptin (ng/mL)	1.000	-	-	-	-	-	-	-	-	-
Proteinuria (mg/day)	0.346**	1.000	-	-	-	-	-	-	-	-
SBP (mmHg)	0.171*	0.095	1.000	-	-	-	-	-	-	-
DBP (mmHg)	0.216**	0.257**	0.273**	1.000	-	-	-	-	-	-
U-osmolarity (mOsm/kg)	0.430**	0.132	0.097	0.070	1.000	-	-	-	-	-
U-volume (mL)	−0.030	−0.036	−0.102	−0.187**	0.001	1.000	-	-	-	-
cIMT (mm)	0.643**	0.268**	0.088	0.274**	0.236**	−0.028	1.000	-	-	-
LVM (g)	0.118	0.055	0.158	0.089	0.085	0.099	0.128	1.000	−0.145	-
LVEDD (mm)	−0.069	0.142	0.094	0.166	0.022	0.128	0.056	0.133	1.000	-
eGFR (ml/min/1.73 m^2^)	−0.304**	−0.386**	−0.253**	−0.272**	−0.204**	0.063	−0.355**	−0.162	0.137	1.000

*p < 0.05

**p < 0.01

**Table 4:** Univariate and multivariate models analysing the correlations between changes (Δ) in PWV, eGFR, copeptin, cIMT and FMD in ADPKD patients

Variable	ΔPWV		ΔFMD		ΔeGFR		Δcopeptin		ΔcIMT	
	Univariate	Multiple	Univariate	Multiple	Univariate	Multiple	Univariate	Multiple	Univariate	Multiple
ΔBMI (kg/m^2^)	0.056(0.429)	-	−0.053(0.457)	-	0.083(0.243)	-	0.019(0.685)	-	0.133(0.054)	-
ΔCalcium (mg/dl)	0.206(0.003)	-	0.011(0.881)	-	−0.038(0.595)	-	0.134(0.200)	-	0.063(0.420)	-
ΔPhosphorus (mg/dl)	0.113(0.112)	-	0.027(0.708)	-	0.117(0.100)	-	0.109(0.150)	-	0.055(0.460)	-
ΔHDL Cholesterol (mg/dl)	0.117(0.099)	-	−0.149(0.035)	−0.175(0.002)	0.071(0.319)	-	0.085(0.365)	-	−0.048(0.484)	-
ΔLDL Cholesterol (mg/dl)	0.260(<0.001)	-	−0.037(0.604)	-	−0.016(0.827)	-	0.053(0.460)	-	−0.038(0.594)	-
ΔTotal Cholesterol (mg/dl)	−0.111(0.118)	-	−0.143(0.044)	-	0.056(0.432)	-	0.091(0.332)		−0.056(0.360)	-
ΔTriglyceride (mg/dl)	0.170(0.016)	-	0.194(0.006)	0.146 (0.010)	−0.101(0.154)	-	−0.107(0.102)	-	0.099(0.163)	-
Δ Hemoglobin (g/l)	−0.008(0.910)	-	−0.030(0.675)	-	0.081(0.256)	-	0.080 (0.134)	-	0.079(0.283)	-
ΔGlucose (mg/dl)	0.060(0.398)	-	−0.075(0.293)	-	−0.127(0.073)	-	−0.050 (0.705)	-	0.117(0.110)	-
ΔAverage 24-h SBP, mmHg	0.128(0.026)	0.249(0.001)	0.366(0.005)	0.268(0.017)	−0.429(<0.001)	−0.348(<0.001)	0.383(<0.001)	-	0.317(<0.001)	-
ΔAverage 24-h D BP, mmHg	−0.018(0.799)	-	−0.180(0.011)	-	0.079(0.263)	-	0.493(<0.001)	0.313(<0.001)	0.385(<0.001)	0.142(0.030)
ΔProteinuria (mg/day)	0.045(0.523)	-	−0.170(0.016)	-	0.060(0.400)	-	0.168(0.018)	0.133(0.018)	0.168(0.017)	0.131(0.028)
Δ CIMT(mm)	0.138(0.051)	-	−0.302(<0.001)	-	−0.065(0.364)	-	0.562(<0.001)	0.347(<0.001)	-	-
Δ Copeptin (ng/mL)	0.179(0.011)	0.267(<0.001)	0.599(<0.001)	0.582(<0.001)	−0.414(<0.001)	−0.331(<0.001)	-	-	0.562(<0.001)	0.391(<0.001)
ΔHs-CRP (mg/l)	0.120(0.034)	-	0.255(<0.001)	-	−0.182(0.009)	-	0.378(<0.001)	0.196(0.001)	0.372(<0.001)	0.210(0.001)

Values are expressed as standardized correlation coefficients (*p* values)

### Multivariate modelling

We explored the observed associations using multiple regression analyses including baseline factors associated with copeptin at *p* < 0.10 in univariate analyses (PWV, FMD, LVM, Proteinuria, U-osmolarity, U-volume, cIMT, LVEDD) (Table 3). We also performed regression analyses of changes over time (Table 4). Of the variables associated with change in eGFR (total model coefficient; *p* < 0.01), SBP (coefficient -0.35; *p* < 0.001) and copeptin (-0.33; *p* < 0.001) emerged as independent. Similar values were obtained for predictors of PWV and FMD, which was also predicted by changes in blood cholesterol and triglycerides. Regarding cIMT, DBP, proteinuria, copeptin and CRP emerged were found as independent predictors (total model coefficient; *p* < 0.001). Also, DBP, proteinuria, CRP and cIMT variables were found to be the most essential variables in predicting Δ copeptin (total model coefficient; *p* < 0.001, Table 4).

### Baseline copeptin as a predictor of future changes

We optimized the positive predictive value of copeptin by performing separate ROC-analyses for each of the CVD surrogates (Table 5). Using these cut-offs, we next applied separate Logistic Regression Models to assess the predictive value of a plasma copeptin above or below these cut-offs for the change (Δ) in the outcome variable until follow-up (Table 5). Finally, multivariate modeling using only baseline parameters was performed to assess the independence of copeptin as a predictor of future changes in CVD markers (Table 6).

**Table 5:** Statistical usefullness of serum copeptin levels as a marker of a FMD, PWV, eGFR, or CIMT above or below the variable median for the cohort at baseline

Variable	Area under curve		Diagnostic statistics				
	AUC	*p*	Co-peptin cut-off used (from ROC)	SEN (%)	SPE (%)	PPV(%)	NPV(%)
FMD (> or ≤7.1%)	0.75(0.68–0.80)	<0.001	0.59 ng/mL	88.0(79.6–93.6)	57.4(47.5–66.9)	63.8(54.8–72.1)	84.9(74.6–92.2)
PWV (> or ≤7.8 m/sec)	0.58(0.50–0.65)	0.064	0.76 ng/mL	51.1(40.4–61.7)	71.3(61.8–79.6)	60.8(48.5–71.2)	63.1(53.9–71.7)
eGFR(> or ≤59 mL/min/1.73 m^2^)	0.61(0.54–0.68)	0.008	0.81 ng/mL	43.5(33.2–55.2)	60.2(50.3–60.9)	52.2(41.4–62.9)	59.1(49.3–68.4)
cIMT (> or ≤0.8 mm)	0.86(0.80–0.90)	<0.001	0.58 ng/mL	79.4(69.6–87.1)	88.9(81.4–94.1)	85.9(76.6–92.5)	83.5(75.4–89.7)

*AUC* Area under curve, *SEN* Specificity, *SPE* Specificity, *PPV* Positive predictive value, *NPV* Negative predictive value

**Table 6:** Multivariate modeling to assess the independence of plasma copeptin as a marker of changes in cardiovascular function and eGFR

Variables	ΔFMD		ΔPWV		ΔeGFR		ΔcIMT	
	Univariate OR(95%CI)	Adjusted OR(95%CI)	Univariate OR(95%CI)	Adjusted OR(95%CI)	Univariate OR(95%CI)	Adjusted OR(95%CI)	Univariate OR(95%CI)	Adjusted OR(95%CI)
ΔLDL (mg/dl)	−0.109(0.125)	-	−0.179(0.011)	−0.162(0.018)	0.007(0.925)	-	0.072(0.311)	-
ΔAverage 24-h SBP, (mmHg)	−0.167(0.018)	-	0.015(0.828)	-	0.112(0.115)	-	0.017(0.810)	-
ΔAverage 24-h D BP,(mmHg)	−0.061(0.394)	-	0.007(0.917)	-	0.104(0.142)	-	0.055(0.442)	-
ΔFMD (%)	-	-	−0.163(0.021)	-	0.029(0.688)	-	−0.197(0.005)	-
ΔPWV (m/sec)	−0.106(0.135)	-	-	-	0.041(0.565)	-	−0.177(0.012)	−0.162(0.009)
ΔeGFR(ml/min/1.73 m^2^)	−0.223(0.001)	−0.252(<0.001)	−0.265(<0.001)	−0.253(<0.001)	-	-	−0.448(<0.001)	−0.473(<0.001)
ΔcIMT (mm)	−0.334(<0.001)	-	−0.024(0.737)	-	−0.188(0.008)	−0.188(0.008)	-	-
ΔCopeptin (ng/mL)	−0.431(<0.001)	−0.448(<0.001)	0.014(0.839)	-	−0.109(0.125)	-	−0.238(0.001)	−0.240(<0.001)

*OR* Odds ratio, *CI* Confidence interval

## Discussion

In this study we measured common surrogate markers of hypertensive (PWV and LVM), atherosclerotic (cIMT) and endothelial (warm ischemia induced FMD) CVD in early-stage ADPKD. All analyses were repeated after a mean 36 months, and we related the results both to each other and to serum levels of the marker of AVP-activity, copeptin. Our data demonstrates that signs of cardiovascular dysfunction, especially of the endothelium, develop already in ADPKD patients with preserved renal function and progresses at a more rapid rate than that of the decline in renal function (assessed as eGFR). We also found that serum levels of copeptin are elevated already before a decline in eGFR in these patients, and that the degree of elevation correlates with functional markers of CVD and also predict a future decline in GFR as well as in FMD.

The most important finding of the present study is the characterization of the vascular dysfunction that appears at an early stage of ADPKD and that may be related to the hypertension commonly seen in these patients. Based on our findings, vascular dysfunction in ADPKD is related to endothelial function (FMD) and potentially atherosclerosis (cIMT) more than to fluid overload (PWV) or cardiac failure (echocardiography). We [16, 17] and others [18, 19] have previously reported an impaired vascular function during early-stage ADPKD in cross-sectional studies and single modalities assessing vascular functions, while Peterson et al. [20] studied endothelium-dependent vasodilation in a rat model of ADPKD and reported changes there that preceded a rise in mean arterial pressure or drop in GFR. With the present data, these findings are extended and broadened, demonstrating the consistent nature of ADPKD vasculopathy (early onset, FMD worse than cIMT, which in turn is worse than PWV and precedes any changes in cardiac geometry) as well as the extent to which vascular decline precedes that of GFR and also develops at a faster rate.

Regarding potential mechanisms mediating the observed endothelial dysfunction, Peterson et al. [20] found that administration of L-arginine (a precursor of nitric oxide, NO) was able to restore vascular function in their model, suggesting a key role for NO signaling. Consistent with this finding, Lothioir et al. [21] recently reported that FMD induced by warm-ischemia, but not that induced by NO, is impaired in ADPKD. Studying 21 normotensive patients (eGFR 99 ± 18 mL/min/1.73 m^2^, 36% smokers) they found a maintained ability to dilate vessels following infusion of an NO-donor, while warm ischemia led to a markedly blunted response in patients as compared to controls. Furthermore, infusion of dopamine was able to ameliorate also the warm-ischemia induced FMD in these patients, further strengthening the reported link between ADPKD and impaired dopaminergic signaling reported previously [22, 23]. Barendregt and colleagues [22] studied eight hypertensive ADPKD patients and reported that urinary dopamine excretion is increased at all levels of sodium intake, while stimulation of renal dopamine production was able to normalize renal hemodynamics, making dopamine receptor agonism a potential therapeutic option. Meanwhile Abdul-Majeed and Nauli [23] used transgenic mice to demonstrate an important role for dopamine receptor (DR)5 in regulating endothelial ciliary length and function in close cooperation with PKD1. Indeed the DR5-linked chemo-sensory function of endothelial cell cilia could even alter the sensitivity to fluid-shear stress, providing a mechanistic explanation for the previous observations.

In the present study, hypertension and alterations in vascular functions were predicted by an elevated baseline circulating copeptin, while changes in copeptin closely followed those in SBP, FMD, cIMT, and, to a lesser extent, PWV. This is consistent with the hypothesis that copeptin functions as a more stable marker of AVP release in response to changes in plasma osmolality and other factors. Recently, the role of AVP in ADPKD has received much attention. Studies have linked AVP-signalling through V2 receptors in the distal renal tubules and collecting ducts to a rise in intracellular cAMP-levels [24] that in turn stimulates cyst growth by several mechanisms that may include chloride-driven fluid secretion from proliferating cyst-derived cells [25]. Meijer et al. [26] demonstrated a relationship between serum copeptin levels and total renal volume, albuminuria, renal blood flow and eGFR. Bortien et al. [10] further established an association between a baseline elevation in copeptin levels and a future decrease in GFR; however, this relation was not found to be independent from other risk factors.

The present study confirms and expands the previous literature by establishing a strong and independent longitudinal 3-year association both between copeptin and eGFR changes, as well as link an elevated copeptin to impaired ischemia-mediated FMD. This may offer observational support for the hypothesis that dopaminergic inhibition of AVP-driven AQP2 expression and recruitment to the cell membrane [27] reflects a more general modulating role on AVP-signalling and encourages the investigation of dopamine a potential therapeutic in ADPKD. Indeed, in the 3-years randomized clinical TEMPO 3:4 trial [5], the V2-inhibitor tolvaptan was shown to retard cyst growth and GFR decline in ADPKD-patients, but the price of the drug has been criticized as high [28] while the number of patients that discontinued treatment due to adverse reactions was significantly higher with tolvaptan than with placebo.

A number of limitations of the study design should be acknowledged and kept inmind when interpreting the results. First, we did not perform genetic profiling of the included ADPKD patients and thus have no way of telling whether PKD1, PKD2 or even undescribed mutations caused the phenotype. Secondly, we did not measure kidney volumes in the study subjects either at baseline or follow-up. We are thus unable to relate copeptin levels to radiological signs of disease progression. Finally, this is a cohort study without active interventions, precluding the attribution of causality to the predictive statistical relationships.

## Conclusion

Vascular dysfunction as reflected by FMD and cIMT, but not PWV or an altered cardiac geometry, precede most other signs of disease in ADPKD but is predicted by elevated levels of the circulating AVP-marker copeptin.

## Abbreviations

ADPKD: Adult polycystic kidney disease
AVP: Vasopressin
cIMT: carotid intima media thickness
CVD: Cardiovascular disease
DBP: Diastolic blood pressure
ELISA: Enzyme-linked immunosorbent assay
FMD: Flow-mediation dilatation
GFR: Glomerular filtration rate
NO: Nitric oxide
PWV: Pulse wave velocity
SBP: Systolic blood pressure
